# Beliefs and perceptions regarding cervical cancer and screening associated with Pap smear uptake in Johannesburg: A cross-sectional study

**DOI:** 10.1371/journal.pone.0246574

**Published:** 2021-02-10

**Authors:** Mantwa Chisale Mabotja, Jonathan Levin, Mary Kawonga

**Affiliations:** 1 School of Public Health, Faculty of Health Sciences, University of Witwatersrand, Johannesburg, South Africa; 2 Gauteng Provincial Health Department, Charlotte Maxeke Johannesburg Academic Hospital, Johannesburg, South Africa; Rudjer Boskovic Institute, CROATIA

## Abstract

**Background:**

Cervical cancer is a major global public health concern, with 85% of cases occurring in low- and middle-income countries. In South Africa, it is the second most common cancer amongst women. Screening and treatment of cervical cancer precursor lesions is associated with a lower incidence and mortality. This research determines the associations between women’s beliefs about cervical cancer and screening and the uptake of Papanicolaou (Pap) smears in Johannesburg, where cervical screening uptake is suboptimal.

**Methods:**

This research was approved by the University of Witwatersrand Human Research Ethics Committee (Medical), clearance certificate number: M170243 and the Johannesburg District Heath Research Committee prior to conducting the study. All participants signed a consent form prior to participating in this study. This cross-sectional analytical study used an interviewer-administered validated measurement scale based on the Health Belief Model (HBM) to describe health beliefs regarding cervical cancer and screening among 280 women aged 30 years and older, attending Johannesburg primary care facilities in 2017. Logistic regression models, with robust estimation of variance to account for clustering of women within clinics, were fitted to identify health beliefs (perceived susceptibility, severity, barriers and benefit, cues to action, and self-efficacy) associated with ever having had a Pap smear (screening uptake), while controlling for knowledge of screening and potential confounders.

**Results:**

Of the 280 women, 177 (63.2%) had ever been screened, 180 (64.3%) were never married, 199 (71.1%) attained secondary education and 133 (47.5%) were employed full time. Women of older age (AOR = 1.6 for a 5-year increase in age; CI: 1.3–1.9; P<0.001), with higher knowledge scores (AOR = 2.5 for a 5-point increase in knowledge score; 95% CI:1.0–6.3;P = 0.051), with lower perceived barriers scores (AOR = 0.4 for a 5-point increase in barriers score; 95% CI:0.3–0.5; P<0.001) and higher perceived severity scores (AOR = 1.3 for a 5-point increase in severity score; 95% CI:1.0–1.6; P = 0.017) were more likely to have had a Pap smear.

**Conclusions:**

This study shows that women who take up screening are older, more knowledgeable regarding cervical cancer and screening, less likely to perceive screening barriers, and more likely to perceive cervical cancer as a severe disease. This highlights that for public health interventions to increase screening uptake, the focus should include tailored behaviour change communication strategies that address women’s beliefs regarding screening barriers and emphasize the severity of cervical cancer.

## Introduction

Cervical cancer is a major public health concern globally and a significant cause of mortality and morbidity in women [1]. It is the 4th most common cancer amongst women globally, with an estimated 311,000 deaths and 570,000 new cases reported worldwide in 2018, with about 85% of these cases occurring in low- to middle-income countries (LMICs) [1].

Cervical cancer is preventable by early detection through screening, and early diagnosis and effective treatment of precancerous lesions [2]. At a population level, prevention through screening is realised only if high screening coverage is achieved, which means a high proportion of eligible women receiving screening tests [2]. High cervical screening coverage is recognised as the most important measure in achieving reductions in incidence and mortality [3]. Screening coverage amongst eligible women in low- to middle-income countries (LMICs) is on average 19%, compared to 63% in high income countries (HICs) [4]. Successful reductions in mortality and morbidity noted in HICs are mainly attributed to increased uptake of screening, due to greater access to healthcare and high level of awareness of screening among women [2].

### Cervical screening coverage

The literature identifies many factors associated with poor coverage of cervical screening, categorised as healthcare provider, health system and personal factors [5]. Health system factors contributing to low screening coverage in LMICs include low investment in screening mainly due to competing healthcare priorities, weak health systems, insufficient financial resources, and limited number of health providers [3]. In South Africa, health system and health provider factors include: lack of leadership and good stewardship of the health system, poor monitoring and evaluation, competing health priorities, poor accessibility and quality of screening services, insufficient human and material resources to perform Pap smears [5–8], as well as poor health provider’s knowledge of, and negative attitudes regarding, cervical screening policy requirements [8, 9].

Personal factors refer to issues such as knowledge, beliefs, and attitudes, which may impact on women’s uptake of available health services. Research available in South Africa on personal factors mostly explores women’s knowledge and attitudes [10–12]. For example, in rural South Africa, Hoque et al [12] showed that low uptake of screening is associated with a low level of knowledge of risk factors and prevention of cervical cancer. Qualitative studies conducted in SA [13, 14] highlight that women’s beliefs such as fear of cancer and misconceptions that Pap smears are associated with “cleaning of the womb” may influence women to not have Pap smears. However, there is a dearth of research exploring the influence of personal factors, such as women’s health beliefs, on cervical screening uptake in South Africa. Such research is useful to inform demand generation strategies to promote appropriate health seeking behaviour amongst eligible women.

### Health Belief Model

The Health Belief Model (HBM) is one of the most widely utilized and oldest models, in which theory from behavioural sciences is applied to understand health behaviours [15]. It is “a psychological health behaviour change model suggesting that people’s beliefs, perceptions and attitudes about a disease determines their willingness to use preventative interventions such as screening for disease” [16] (p25). This model was introduced in the 1950s initially by Hochbaum et al, and consisted of four main constructs: perceived susceptibility, barriers, severity and benefits [17–19]. The HBM describes an individual’s beliefs regarding the likelihood of experiencing a condition or disease that could adversely affect their health (perceived susceptibility) [20]; an individual’s interpretation of the degree of severity of a disease (perceived severity) [20], one’s beliefs that using the prevention service will benefit the individual, e.g. by preventing disease (perceived benefit) [18, 19]; and factors perceived to hinder one’s adoption of healthy behaviours, for example, cost or convenience of the service (perceived barriers) [18, 19]. Two other constructs were added later to the HBM [18, 19]—cues to action (a cue or trigger that prompts one’s engagement in health-promoting behaviours [21], for example, information from health care providers, the media, or close others) and self-efficacy (perceived confidence in one’s capability to adopt behaviour that leads to desired favourable outcome) [18, 19, 22].

Measurement scales based on the HBM have been used to understand various health-seeking behaviours. For example, Champion developed HBM instruments to explore breast cancer screening uptake [18, 23, 24]. Champion’s Health Belief Model (CHBM) and Champion’s Self-efficacy (CSE) scales have been translated, adapted and found to be reliable and valid for use in various cultures and settings [25–29], and have been adapted and used, though not validated, in the African context in countries such as Botswana and South Africa [30, 31].

This study applies the HBM to describe the beliefs regarding cervical cancer and screening amongst women attending primary care clinics in the Johannesburg district, South Africa, and determines the associations between their beliefs and uptake of Pap smears. The study was guided by the hypothesis that health beliefs regarding Pap smears differed between women who had ever had a Pap smear (uptake) and those who had not.

## Methods

### Study design and setting

This research was approved by the University of Witwatersrand Human Research Ethics Committee (Medical), clearance certificate number: M170243 and the Johannesburg District Heath Research Committee prior to conducting the study. All participants signed a consent form prior to participating in this study. This cross-sectional study with an analytical component was conducted in Johannesburg, one of the wealthiest districts in South Africa [32]. The Johannesburg district is divided into seven health sub-districts named from A to G. Within sub-districts, the public sector health facilities at primary healthcare level include clinics, community healthcare centres (CHCs) and district hospitals. Clinics and CHCs, which are administered by either local or provincial government health authorities, provide free cervical screening services (Pap smears) for HIV positive women of any age at HIV diagnosis and for HIV negative women from age 30. The cervical screening coverage for health districts in South Africa is measured by the number of cervical smears taken in women 30 years and older as a proportion of the female population 30 years and older factored for one smear every 10 years [33]. In this district (study site) it was 47,4% during 2016 to 2017 [33, 34] significantly lower than the district (55%), provincial (55%) and national (62%) targets.

### Sampling and sample size considerations

A two-stage sampling procedure was used. At the first stage, in each sub-district two primary care facilities were randomly selected by sampling with probability proportional to clinic size. We used the clinic weekly head count (number of adult attendees presenting to the clinic for any services) as a measure of clinic size. Since not all sub-districts have a CHC or district hospital, only clinics were included in the study.

In the second stage of sampling, we selected a systematic sample of 20 women per clinic. The systematic sample was selected by sampling the first participant randomly from the women waiting in the queue at the clinic, and thereafter sampling every 4th woman until the required sample size of 20 women was reached. This led to a total sample size of 280 women clustered within 14 clinics. The clustering of women within clinics results in a loss of precision compared to a simple random sample of 280 women. This loss of precision is expressed using the design effect or deff. We assumed a moderate design effect of 1.5, which can be interpreted as our sample achieving the same precision as that of a simple random sample of size 280/1.5 or approximately 190 women; this is the effective sample size. An effective sample size of 190 women would enable the proportion of women who have ever had a Pap smear to be estimated with a precision of ±7.3%. In addition the study had over 80% power to detect as statistically significant at the 5% level an absolute difference of 20% in the proportion of women who have had a Pap smear between two equally sized groups (for example those with a high score on one of the HBM scales and those with a low score on the scale). Since in fact we used the actual scores on the HBM constructs rather than dichotomizing the score, this increased the power to detect an association between a particular HBM construct and having had a Pap smear.

The study had a 100% response rate. Within each sampled clinic, eligible women were women aged 30 or older, attending the clinic on the day of the study for any health service (including Pap smear) in 2017. The age restriction was consistent with the target age group for screening as stated in the national cervical cancer prevention and control policy [35]. A history of cervical cancer and HIV status were not included among the selection criteria.

### Data collection and variables

**Fig 1:**
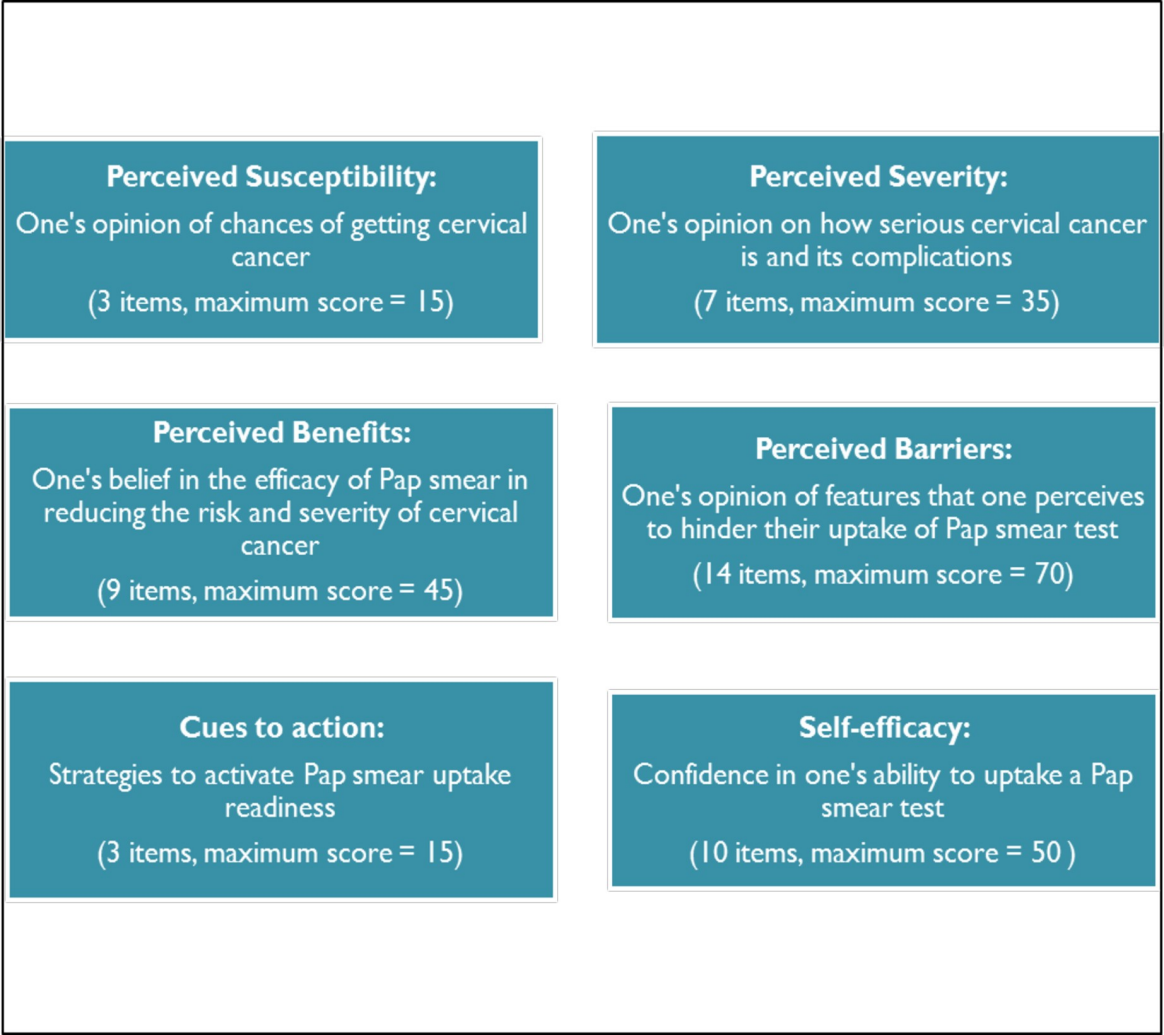
Health Belief Model constructs measured in the study.

Following a pilot study, data were collected from June to August 2017, using an interviewer-administered structured questionnaire. The researcher and a trained research assistant administered the questionnaire. The questions were in English and the interviewers interpreted phrases into local languages (Isizulu or Sesotho) during the interview where the respondent could not understand English. Participant responses were captured directly onto REDCap software [36] during the interview. Data were collected in each clinic, returning every day until the required number of participants had been interviewed, before moving to the next clinic. The questionnaire measured the six HBM constructs (explanatory variables), and Pap smear uptake (the outcome binary variable recording whether or not the respondent had ever had a Pap smear). The HBM constructs were measured using the adapted and modified CHBM and CSE scales [20, 29]. Items in each construct were measured using five-point Likert scales ranging from strongly disagree or less positive (1 point) to strongly agree or more positive beliefs (5 points). See Fig 1.

Since the HBM refers to modifying factors that may influence behaviours–including age, socio-economic status and general knowledge about cervical cancer and screening [21], we included questions on age, marital status, employment status, highest level of education attained in our questionnaire as well as questions on level of knowledge about screening and cervical cancer. The cervical cancer knowledge questions included knowing what cervical cancer is, risk factors for cervical cancer, as well as signs and symptoms of cervical cancer and treatment options. The Pap smear knowledge questions include knowing what Pap smear is, how it is performed, whether the participants knew the existing South African screening policy and the recommended age to start cervical screening test. Each correct answer was assigned a score of one while an incorrect answer was scored zero, with the knowledge score calculated as the sum of the score for knowledge regarding cervical cancer and the score for the knowledge regarding Pap smears. The questions were adapted and modified from an existing validated tool which measures knowledge of screening and cervical cancer [37]. While the tool has been validated in other numerous settings, it has not been validated in the African setting. During the interview, following the knowledge questions, the researcher explained the concepts of cervical cancer and Pap smears before continuing with the HBM construct section of the questionnaire.

### Data management and analysis

The data were captured in a REDCap database and analysed in STATA release 13.0 [38]. All analyses took into account the clustering of women within clinics through the robust estimation of standard errors, achieved using the Stata commands for the analysis of survey data, which also accounted for the stratification by sub-district. For each scale (the six HBM scales and the one knowledge scale) the item scores were summed to create a score. Continuous variables (age, knowledge score and the six HBM construct scores) were summarized using means and 95% confidence intervals, overall and by whether or not the woman had ever had a Pap smear. Logistic regression models with robust estimation of standard errors were fitted with “ever had a Pap smear” as the outcome variable and one of the explanatory variables at a time, adjusting for age, in order to see whether there was any evidence of an association between the variable and having had a Pap smear. Thereafter a multiple logistic regression model was fitted with all six HBM constructs, the knowledge score, age and any additional variables that were significant in the single age-adjusted models. Variables that were not significant at the 5% level in the full model were sequentially removed. In order to interpret the effects of the continuous variables more easily, we considered a 5-pointincrease in each scale or a 5-year increase in age. Interactions were considered one by one between the HBM constructs in the model and the confounding variables that remained in the model (in this case age and knowledge score).

## Results

As shown in Table 1, of the 280 women in the study, 177 (63.2%) had ever had at least one Pap smear in their lifetime, 180 (64.3%) were never married and 199 (71.1%) had secondary education. The mean age of participants was 40.4 [95% CI 39.3–41.4]. Compared to women who had never had a Pap smear, screened women had a higher mean age (43.1 [95% CI 41.8–44.4] vs 35.7 [95% CI 33.7–37.7]), higher mean knowledge score (13.9 [95% CI 13.6–14.3] vs 12.5 [95% CI 11.7–13.3]) and were more likely to be employed full time (96 (54.24%) vs 37 (35.9%)). Of the 280 women, 111 (42.1%) gave a correct response on what cervical cancer is and 173 (61.8%) gave a correct response on what Pap smear is. There were no differences in marital status and education level between screened and unscreened women (Table 1).

**Table 1:** Socio-demographic factors and knowledge scores by Pap smear history.

Variables	All women (N = 280) *n (%)*	Ever had a Pap smear *(N = 177)* *n (%)*	Never had a Pap smear *(N = 103)* *n (%)*
Marital status			
Single/never married	180 (64.3)	112 (63.3)	68 (66.0)
Married/living with a partner	80 (28.6)	51 (28.8)	29 (28.2)
Divorced/Separated/widowed	20 (7.1)	14 (7.9)	6 (5.8)
Education level			
≤Primary school	32 (11.4)	19 (10.7)	13 (12.6)
Secondary school	199 (71.1)	132 (74.6)	67 (65.1)
Tertiary education	49 (17.5)	26 (14.7)	23 (22.3)
Employment status			
Employed full-time	133 (47.5)	96 (54.2)	37 (35.9)
Employed part-time	32 (11.4)	19 (10.7)	13 (12.6)
Self-employed	8 (2.9)	2 (1.1)	6 (5.8)
Unemployed	107 (38.2)	60 (33.9)	47 (45.6)
Age			
Mean [95% CI]	40.4 [39.3–41.4]	43.1 [41.8–44.4]	35.7 [33.7–37.7]
Total knowledge score			
Mean [95% CI]	13.4 [12.9–13.9]	13.9 [13.6–14.3]	12.5 [11.7–13.3]

Table 2 shows that screened women had lower mean HBM perceived barriers scores than unscreened women (19.6 [95% CI 18.5–20.7] vs 25.9 [95% CI 23.9–28.1]). The two groups of women had similar mean HBM scores for perceived susceptibility (10.5 [95% CI 9.3–11.6] vs 11.1 [95% CI 9.7–12.5]), perceived severity (23 [95% CI 20.7–25.3] vs 21.8 [95% CI 19.9–23.8]), perceived benefits (41.7 [95% CI 41.2–42.3] vs 41.3 [95% CI 39.9–42.6]), cues to action (8.4 [95% CI 7.5–9.3] vs 7.5 [95% CI 6.4–8.7]) and self- efficacy (49.4 [95% CI 49.0–49.8] vs 48.2 [95% CI 46.8–49.6]).

**Table 2:** Health Belief Model construct scores according to Pap smear history.

Characteristics	Total (N = 280) Mean [95% CI]	Ever had a Pap smear *(N = 177)* Mean [95% CI]	Never had a Pap smear *(N = 103)* Mean [95% CI]	*p-value*
Susceptibility score	10.7 [9.5–11.9]	10.5 [9.3–11.6]	11.1 [9.7–12.5]	0.130
Severity score	22.6 [20.6–24.5]	23 [20.7–25.3]	21.8 [19.9–23.8]	0.235
Benefits score	41.6 [40.9–42.2]	41.7 [41.2–42.3]	41.3 [39.9–42.6]	0.414
Barriers score	21.9 [20.2–23.7]	19.6 [18.5–20.7]	25.9 [23.9–28.1]	0.000
Cues to action score	8.1 [7.2–9.0]	8.4 [7.5–9.3]	7.5 [6.4–8.7]	0.091
Self-efficacy score	48.9 [48.2–49.7]	49.4 [49.0–49.8]	48.2 [46.8–49.6]	0.051

The logistic regression model which looked at one variable at a time adjusted for age found that knowledge score, the perceived barriers and perceived self-efficacy scores were associated with the uptake of Pap smears (Table 3).

**Table 3:** Association between HBM constructs and Pap smear uptake, adjusting for potential confounding variables.

Characteristics	*OR adjusted for age [95% CI]*	*p-value*	*Adjusted OR [95% CI]*	*p-value*
Marital status Single/never married (reference) Married/living with a partner Divorced/Separated/Widowed	Ref 0.8 [0.3–1.7] 0.6 [0.1–3.8]	0.757		
Education level ≤Primary school (reference) Secondary school Tertiary education	Ref 3.9 [0.8–20.4] 3.2 [0.6–16.6]	0.242		
Employment status Employed full-time (reference) Employed part-time Self-employed Unemployed	Ref 0.4 [0.2–1.1] 0.1 [0.03–0.6] 0.5 [0.2–1.0]	0.138		
Age	1.7 [1.3–2.1]	<0.001*	1.6 [1.3–1.9]	<0.001*
Knowledge score	3.9 [1.3–11.5]	0.019*	2.5 [1.0–6.3]	0.051**
Susceptibility score	0.9 [0.6–1.2]	0.389	0.7 [0.4–1.1]	0.1787
Severity score	1.2 [0.9–1.5]	0.199	1.3 [1.0–1.6]	0.017*
Benefits score	1.2 [0.6–2.3]	0.509	1.0 [0.7–1.6]	0.899
Barriers score	0.4 [0.3–0.6]	<0.001*	0.4 [0.3–0.5]	<0.001*
Cues to action score	1.3 [0.8–2.3]	0.266	1.3 [0.7–2.2]	0.415
Self-efficacy score	2.1 [1.3–3.3]	0.009*	1.2 [0.7–2.1]	0.572

* P value< 0.05

**P value <0.1

When controlling for the modifying variables which were statistically significant at the 5% level in the univariable model (age, knowledge, perceived barriers and self-efficacy scores), the multivariable logistic regression showed that independent factors associated with Pap smear uptake were age, perceived severity score and perceived barriers score, while knowledge score was marginally significant. Women with higher knowledge scores (AOR = 2.5 for a 5-point increase in knowledge score; 95% CI:1.0–6.3;P = 0.051), older age (AOR = 1.6 for a 5-year increase in age; 95% CI: 1.3–1.9; P<0.001), lower perceived barriers scores (AOR = 0.4 for a 5-point increase in barriers score; 95% CI:0.3–0.5; P<0.001) and higher perceived severity scores (AOR = 1.3 for a 5-point increase in severity score; 95% CI:1.0–1.6; P = 0.017) were more likely to have had a Pap smear (see Table 3). Interactions between barrier scores, knowledge, severity scores and age were investigated but none was significant at the 5% level.

## Discussion

This study used the HBM to describe associations between health beliefs regarding cervical cancer and screening and uptake of Pap smears amongst women attending health clinics in Johannesburg.

Our study found that women who perceive more barriers to accessing Pap smear tests were less likely to have had a Pap smear than those who perceive fewer barriers, and that women’s negative perceptions of the Pap smear test and the health services, influence screening uptake in the study context. The high-scoring perceived barriers included: fear of having abnormal cells detected in the cervix, being ashamed to open legs and show private parts, expectations of female health providers performing the test not being met, and perceptions such as the test being painful and taking long to perform. It was beyond the scope of this study to explore these underlying socio-cultural barriers, but further qualitative studies are recommended to explore these barriers in Johannesburg. Our findings are consistent with studies in South Africa which found that barriers to cervical screening include fear of a bad result, the woman’s partner resisting cervical screening, the test being too painful, cost barriers and lack of information about the disease [14, 39]. Perceived barriers highlighted in studies elsewhere include barriers relating to fears and other psychological concerns, health services, socio-cultural norms and stigma [40, 41].

Our study also shows that women who perceived cervical cancer to be a severe disease were more likely to have had a Pap smear. Women who agreed that cervical cancer is a terminal illness which they feared, who admitted that the thought of cervical cancer scares them, who were afraid of developing and living with cervical cancer, and who believed death from cervical cancer is not rare, were more likely to have had a Pap smear than those who scored lower on these beliefs. This finding is consistent with a study conducted in Botswana [42], which found that a significant number of women agreed that cervical cancer is as severe as other cancers, that it makes a woman’s life difficult and causes infertility [42].

We also found that having a higher level of knowledge regarding cervical cancer and screening increases the likelihood of having had a Pap smear. Low knowledge and awareness of cervical cancer signs and symptoms, causes, risk factors, prevention and treatment options as well as low knowledge on screening have been reported in studies conducted in South Africa [39, 43], as has the observation that a lack of knowledge about cervical cancer and screening hinders the uptake of screening interventions.

Our findings on the association between perceived barriers and screening uptake suggest the need for public health interventions that include tailored behaviour change communication strategies which are sensitive to socio-cultural beliefs and aim to clear misconceptions about the screening test, as well as health providers changing how they provide care to meet women’s expectations. Women are more likely to take-up Pap smears if they perceive the cost to be reasonable and there are no barriers to having a Pap smear [39]. As a study conducted in Mexico showed, uptake of screening can be increased by addressing perceived barriers through strategies such as community outreach programs by lay health workers who speak the local language, distribution of materials printed in the local language, and using culturally-sensitive media [41].

Our study findings also suggest that increasing screening uptake in Johannesburg requires public health interventions that focus on changing women’s perceptions regarding the severity of cervical cancer. As the HBM postulates, the likelihood of a person engaging in a health-seeking behaviour is based on their own understanding of the degree of severity of a disease [17]. To identify appropriate interventions that enable women in Johannesburg to internalise the belief that cervical cancer is a severe condition, lessons can be learned from a study conducted in urban areas of Lagos [44] which showed that a community-based educational campaign disseminating language-appropriate and culturally sensitive educational information could reach high risk women and increase their knowledge of cervical cancer and screening. That study however did not assess change in screening behaviour. Research evidence from other settings is however available showing various educational interventions that are effective at increasing awareness as well as screening uptake, including home-based provision of educational information one-on-one by lay health workers [44] and media-based messaging combined with community outreach by lay health providers [45]. Further, a 2018 systematic review identified various health education methods effective at changing women’s cervical screening behaviour (including pamphlets, mailed messages, group discussions, amongst others) [46]. That review however did not include studies assessing the effectiveness of population-based health education programmes, and did not find any studies in Sub-Saharan Africa [46].

Experiences in South Africa imply that mass media health education campaigns and behaviour change strategies may be useful, and that health authorities implementing such interventions to increase uptake of cervical screening must concentrate on achieving high levels of exposure to socially-relevant health messages. For example a social and behaviour change communication edutainment intervention in South Africa named ‘Soul City’, a mass media intervention that utilises radio dramas, photo-comics and television dramas to address issues such as HIV, cervical cancer, domestic violence and many others, has been extensively evaluated and has been shown to facilitate supportive change or maintain safe sexual behavioural practices [47–49]. Such behaviour change interventions have high levels of exposure and ability to reach high numbers of at risk women. Further, research conducted in a South African peri-urban community that evaluated the effectiveness of two media interventions (radio-drama and photo-comic) for increasing cervical screening uptake found that only the radio-drama was effective, and its effectiveness was limited only by low levels of exposure [50]. Research in South Africa evaluating methods for successfully changing women’s health beliefs and cervical cancer screening behaviors is lacking, but it is needed to inform the design and content of behavior change strategies relevant for this context.

As far as the authors are aware, this is the only study describing women’s beliefs regarding cervical cancer and Pap smears using the HBM in the Johannesburg district where cervical screening coverage is low. Our research identifies health beliefs that are potential targets for interventions to improve screening uptake amongst women over 30 years of age in the district. There are however some limitations related to our study. The participants were women attending clinics; hence it is difficult to generalize the findings to women in the community who are not attending clinics, and women attending health services at other levels of care in the district such as community health centres and district hospitals. The cross-sectional study design means that temporality cannot be determined (whether women’s beliefs and perceptions preceded the uptake of screening). The study did not collect medical history data and did not exclude women with history of cervical cancer, this is a potential selection bias as women with cervical cancer might have different perceptions regarding cervical cancer and screening, as compared to women who have no history of cervical cancer. The questionnaire used in this research was interviewer-administered, which could have resulted in socially desirable responses considering the interviewer’s presence (information bias), however, a self-administered questionnaire could have resulted in incomplete responses and might have been difficult for respondents who are unable to read or write in English to complete.

The scales (CHBM, CSE) and cervical cancer awareness tool adapted to compile the questionnaire for this study have been widely utilized and though validated in various settings, have not been validated in the African context. This may have an impact on the interpretation of the results in the context of South Africa. The questionnaire was written in English and where necessary questions were interpreted on the spot, which could have led to measurement error–inconsistent interviewer interpretations of responses given in other local languages. Finally, a question on HIV status was not included in the questionnaire as this is not addressed in the existing HBM scales. In the context of South Africa where there is a high HIV prevalence, this omission could have resulted in a potential selection bias as HIV positive women are screened more regularly and earlier than age 30. However, including HIV status and medical history (history of cervical cancer) in the questionnaire could have resulted in participants not being willing to participate. A future case control study with age as a matching factor is recommended.

## Conclusion

Cervical cancer remains a significant cause of mortality among women in South Africa, yet it is preventable through screening. Personal factors such as health beliefs may influence screening (Pap smear) uptake. Our study has shown that health beliefs that were independently associated with whether or not a woman had had a Pap smear were two HBM constructs (perceived severity of cervical cancer and perceived barriers to cervical screening), as well as age and knowledge regarding cervical cancer and screening. The findings of this study imply that to increase the screening uptake in Johannesburg district, health authorities should implement tailored behaviour change communication strategies that target women below age forty, include behaviour change messages that consider and are sensitive to socio-cultural beliefs, emphasize the severity of cervical cancer, aim to clear misconceptions about the screening test; and enable health providers to change how they provide care to meet women’s expectations. Qualitative studies are recommended to further explore barriers to cervical cancer screening amongst women in Johannesburg.

## Supporting information

S1 File Dataset. (DTA)

S2 File Data collection tool-questionnaire. (PDF)

## List of abbreviations

CHBM: Champion’s Health Belief Model scale
CHC: Community Health Centre
CSE: Champion’s Self-efficacy scale
DHB: District Health Barometer
HBM: Health Belief Model
HICs: High Income Countries
HIV: Human Immunodeficiency Virus
HPV: Human papilloma virus
Johannesburg District: Johannesburg Metropolitan Health District
LMICs: Low to Middle Income Countries
NDoH: National Department of Health
Pap smear: Papanicolaou smear
PHC: Primary Health Care
SA: South Africa
VIA: Visual inspection with acetic acid
WHO: World Health Organization
