# Supplementary material for: Beliefs and perceptions regarding cervical cancer and screening associated with Pap smear uptake in Johannesburg: A cross-sectional study
Source: PLoS One. 2021 Feb 10;16(2):e0246574. doi: 10.1371/journal.pone.0246574 (PMC7875386; doi:10.1371/journal.pone.0246574)
Supplement: S2 File — (PDF) [file pone.0246574.s002.pdf]

# Beliefs and perceptions regarding cervical cancer and screening associated with Pap smear uptake in Johannesburg

Thank you for taking part in this interview, we appreciate your time and valuable input. There is no right or wrong answer, and please do not feel shy or embarassed to give us your honest answers. This questionnaire will not be longer than 30 minutes.

Today's date: ______________________

Clinic name: ______________________

**We would like to ask you a few questions about yourself. This will help us to analyse the results of the study. The data collected will help us to identify specific age or demographic groups of people who participated in the study. You will not be asked your name and all of your answers will be kept strictly confidential.**

| | Yes | No |
|---|---|---|
| Did you receive the information sheet for this study? | ◯ | ◯ |
| Did you sign the consent form? | ◯ | ◯ |

1. What is your age?

______________________
(only age 30 and older)

2. What is your marital status?

◯ Single/never married
◯ Married/living with partner
◯ Divorced/separated
◯ Widowed
◯ Prefer not to say

3. What is the highest level of education obtained?

◯ No formal education
◯ Primary school
◯ Secondary school
◯ Tertiary education
◯ Prefer not to say
◯ Other

Please specify ______________________

4. Are you currently:

◯ Employed full-time
◯ Employed part-time
◯ Unemployed
◯ Self-employed
◯ Retired
◯ Still studying
◯ Prefer not to say
◯ Other

Please specify ______________________

5. Have you, your family, or close friends had cancer?

◯ No one
◯ You
◯ Partner
◯ Close family member
◯ Other family member
◯ Close friend
◯ Other friend
◯ Prefer not to say

## 6. I would like to know what people know about cervical cancer.

6.1 Briefly explain what is cervical cancer ______________________

6.2 Where did you learn about cervical cancer?

- ○ Media (TV,radio, newspaper, internet)
- ○ Friend
- ○ Community member
- ○ Posters
- ○ Pamphlets
- ○ Health educator
- ○ Health professional
- ○ Never learnt about it
- ○ Other

please specify ______________________

## 7. I would like to know what people know about Pap smears

7.1 Briefly explain what is a Pap smear ______________________

7.2 Where did you learn about Pap smears?

- ◯ Media (TV, radio, newspapers, internet)
- ◯ A friend
- ◯ A community member
- ◯ Health professional
- ◯ Health educator
- ◯ Poster
- ◯ Pamphlet
- ◯ Never learnt about it
- ◯ Other

Please specify ______________________

**8. The following questions are all about cervical cancer, which is cancer of the cervix (sometimes called the opening of the womb). Some questions are about Pap smear test which is a test done for cervical cancer to test for presence of abnormal cells on the opening of the womb.**

| | Yes | No |
|---|---|---|
| Have you had a Pap smear test done in your lifetime? | ○ | ○ |

**9. The following may or may not be true regarding cervical cancer. We are interested in your opinion**

| | True | False | Don't know |
|---|---|---|---|
| Cervical cancer is one of the most common cancers among women | ○ | ○ | ○ |
| Cervical cancer is preventable | ○ | ○ | ○ |
| Sexual transmitted infections increase the risk of cervical cancer among women. | ○ | ○ | ○ |
| Bleeding and spotting after menopause may be associated with cervical cancer | ○ | ○ | ○ |
| Cervical cancer may be without sign in early stages. | ○ | ○ | ○ |

**10. The following may or may not increase a woman's chance of developing cervical cancer. How much do you agree that each of these can increase a woman's chance of developing cervical cancer?**

| | Strongly disagree | Disagree | Not sure | Agree | Strongly agree |
| --- | --- | --- | --- | --- | --- |
| Infection with HPV (human papillomavirus) | ○ | ○ | ○ | ○ | ○ |
| Having many sexual partners | ○ | ○ | ○ | ○ | ○ |
| Having a weakened immune system (e.g because of HIV/AIDS, immunosuppressant drugs or having a transplant) | ○ | ○ | ○ | ○ | ○ |
| Smoking any cigarette at all | ○ | ○ | ○ | ○ | ○ |
| Not going for regular Pap smear tests | ○ | ○ | ○ | ○ | ○ |

11. As far as you are aware, is the department of health providing Pap smear services in South Africa?

○ Yes
○ No
○ I don't know

11.1 If yes, at what age are the women suppose to start screening for cervical cancer in South Africa? ____________________

**12. The following may or may not be true regarding Pap smear test. We are interested in your opinion**

| | True | False | I don't know |
| --- | --- | --- | --- |
| Pap smear test before symptomatic cervical cancer, may help detect cervical cancer earlier | ○ | ○ | ○ |
| Pap smear test is only necessary after age 65 | ○ | ○ | ○ |
| Pap smear test is recommended only for older women. | ○ | ○ | ○ |
| Pap smear test should be performed only if infection and bleeding was seen | ○ | ○ | ○ |

## 13. PERCEIVED SUSCEPTIBILITY

**You may agree or disagree with any of the following statements. Please do state how much do you agree with each of these statements, from strongly agree to strongly disagree.**

| | Strongly disagree | Disagree | Not sure | Agree | Strongly agree |
|---|---|---|---|---|---|
| It is likely that I will get cervical cancer in the future | ○ | ○ | ○ | ○ | ○ |
| My chances of getting cervical cancer in the next few years are high | ○ | ○ | ○ | ○ | ○ |
| I feel I will get cervical cancer some time during my life | ○ | ○ | ○ | ○ | ○ |

## 14. PERCEIVED SEVERITY/SERIOUSNESS

**You may agree or disagree with any of the following statements. Please do state how much do you agree with each of these statements, from strongly agree to strongly disagree.**

| | Strongly disagree | Disagree | Not sure | Agree | Strongly agree |
|---|---|---|---|---|---|
| The thought of cervical cancer scares me | ○ | ○ | ○ | ○ | ○ |
| When I think about cervical cancer, my heart beats faster | ○ | ○ | ○ | ○ | ○ |
| I am afraid to think about cervical cancer | ○ | ○ | ○ | ○ | ○ |
| Problems I would experience with cervical cancer would last a long time | ○ | ○ | ○ | ○ | ○ |
| Cervical cancer would threaten a relationship with my boyfriend, husband, or partner | ○ | ○ | ○ | ○ | ○ |
| If I had cervical cancer my whole life would change | ○ | ○ | ○ | ○ | ○ |
| If I developed cervical cancer, I would not live longer than 5 years | ○ | ○ | ○ | ○ | ○ |

## 15. PERCEIVED BENEFITS AND HEALTH MOTIVATION

**You may agree or disagree with any of the following statements. Please do state how much do you agree with each of these statements, from strongly agree to strongly disagree.**

| | Strongly disagree | Disagree | Not sure | Agree | Strongly agree |
|---|---|---|---|---|---|
| I want to discover health problems early | ○ | ○ | ○ | ○ | ○ |
| Maintaining good health is extremely important to me | ○ | ○ | ○ | ○ | ○ |
| I look for new information to improve my health | ○ | ○ | ○ | ○ | ○ |
| I feel it is important to carry out activities which will improve my health | ○ | ○ | ○ | ○ | ○ |
| If I have a Pap smear test regularly and the result is good, I don't need to worry too much about cervical cancer | ○ | ○ | ○ | ○ | ○ |
| Having regular Pap Smear Tests will help to find changes to the cervix, before they turn into cancer | ○ | ○ | ○ | ○ | ○ |
| If cervical cancer was found at a regular Pap Smear Test its treatment would not be so bad | ○ | ○ | ○ | ○ | ○ |
| I think that having a regular Pap Smear Test is the best way for cervical cancer to be diagnosed early | ○ | ○ | ○ | ○ | ○ |
| Having regular Pap Smear Tests will decrease my chances of dying from cervical cancer | ○ | ○ | ○ | ○ | ○ |

## 16. PERCEIVED BARRIERS

**You may agree or disagree with any of the following statements. Please do state how much do you agree with each of these statements, from strongly agree to strongly disagree.**

| | Strongly disagree | Disagree | Not sure | Agree | Strongly agree |
|---|---|---|---|---|---|
| I am afraid to have a Pap Smear Test for fear of a bad result | ○ | ○ | ○ | ○ | ○ |
| I am afraid to have a Pap Smear Test because I don't know what will happen | ○ | ○ | ○ | ○ | ○ |
| I cannot remember to have a Pap Smear Test regularly | ○ | ○ | ○ | ○ | ○ |
| I don't know where to go for a Pap Smear Test | ○ | ○ | ○ | ○ | ○ |
| Having a Pap Smear Test takes too much time | ○ | ○ | ○ | ○ | ○ |
| I have other problems more important than having a Pap Smear Test in my life | ○ | ○ | ○ | ○ | ○ |
| I would be ashamed to lie on a examination bed and show my private parts to have a Pap Smear Test | ○ | ○ | ○ | ○ | ○ |
| I am too old to have a Pap Smear Test regularly | ○ | ○ | ○ | ○ | ○ |
| There is no health centre close to my house to have a Pap Smear Test | ○ | ○ | ○ | ○ | ○ |
| Having a Pap Smear Test is too painful | ○ | ○ | ○ | ○ | ○ |
| If there is cervical cancer development in my destiny, having a Pap Smear Test cannot prevent it | ○ | ○ | ○ | ○ | ○ |
| Health professionals doing Pap Smear Test are rude to women | ○ | ○ | ○ | ○ | ○ |
| I prefer a female doctor or nurse to conduct a Pap Smear Test | ○ | ○ | ○ | ○ | ○ |
| I will never have a Pap Smear Test if I have to pay for it | ○ | ○ | ○ | ○ | ○ |

## 17. CUES TO ACTION/HEALTH MOTIVATION

**You may agree or disagree with any of the following statements. Please do state how much do you agree with each of these statements, from strongly agree to strongly disagree.**

| | Strongly disagree | Disagree | Not sure | Agree | Strongly agree |
|---|---|---|---|---|---|
| I eat well balanced meals for my health | ○ | ○ | ○ | ○ | ○ |
| I exercise at least 3 times a week for my health | ○ | ○ | ○ | ○ | ○ |
| I have regular health check-ups even when I am not sick | ○ | ○ | ○ | ○ | ○ |

## 18. SELF-EFFICACY

**You may agree or disagree with any of the following statements. Please do state how much do you agree with each of these statements, from strongly agree to strongly disagree.**

| | Strongly disagree | Disagree | Not sure | Agree | Strongly agree |
|---|---|---|---|---|---|
| I can arrange transportation to get a Pap Smear Test | ○ | ○ | ○ | ○ | ○ |
| I can arrange other things in my life to have a Pap Smear Test | ○ | ○ | ○ | ○ | ○ |
| I can talk to people at the Pap Smear centre about my concerns | ○ | ○ | ○ | ○ | ○ |
| I can get a Pap Smear done even if I am worried | ○ | ○ | ○ | ○ | ○ |
| I can get a Pap Smear done even if I don't know what to expect | ○ | ○ | ○ | ○ | ○ |
| I can find a way to pay for a Pap Smear Test | ○ | ○ | ○ | ○ | ○ |
| I can make an appointment for a Pap Smear Test | ○ | ○ | ○ | ○ | ○ |
| I know for sure I can get a Pap Smear Test done if I really want to | ○ | ○ | ○ | ○ | ○ |
| I know how to go about getting a Pap Smear Test | ○ | ○ | ○ | ○ | ○ |
| I can find a place to have a Pap Smear Test | ○ | ○ | ○ | ○ | ○ |

**Cervical cancer is the abnormal growth of cells in the cervix (sometimes called the opening of the womb). The primary cause of cervical pre-cancer and cancer is persistent infection with one or more of the high-risk types of human papillomavirus (HPV).HPV is the most common infection acquired during sexual relations, usually early in sexual life. In most women and men who become infected with HPV, these infections will**
**resolve spontaneously. A minority of HPV infections persist; this may lead to cervical pre-cancer, which, if not treated, may progress to cancer 10 to 20 years later. Women living with HIV are more likely to develop persistent HPV infections at an earlier age and to develop cancer sooner.**

**Cervical pre-cancer can be diagnosed early through regular screening (Pap smear) test. A Pap smear test is a procedure where cells from your cervix are gently scraped away and then examined for abnormal growth in the laboratory. The procedure may be mildly uncomfortable, but does not cause any long-term pain.**

**The department of health in South Africa provides Pap smears in clinics and community health centers. The current policy states that women can have a Pap smear from the age of 30, and every 10 years provided the results are normal, however for HIV positive women the tests are done earlier than the age of 30.**

**For more information, please read the pamphlet given to you and feel free to visit your local clinic.**

Thank you for participating in this study. We greatly appreciate your time
